# The α-Cyclodextrin/Moringin Complex: A New Promising Antimicrobial Agent against *Staphylococcus aureus*

**DOI:** 10.3390/molecules23092097

**Published:** 2018-08-21

**Authors:** Letizia Romeo, Veronica Lanza Cariccio, Renato Iori, Patrick Rollin, Placido Bramanti, Emanuela Mazzon

**Affiliations:** 1 IRCCS Centro Neurolesi “Bonino-Pulejo”, Via Provinciale Palermo, Contrada Casazza, 98124 Messina, Italy; letizia.romeo@hotmail.it (L.R.); veronica_lanzacariccio@yahoo.it (V.L.C.); bramanti.dino@gmail.com (P.B.); 2 Consiglio per la Ricerca in Agricoltura e l’Analisi dell’Economia Agraria, Centro di Ricerca Agricoltura e Ambiente (CREA-AA), Via di Corticella 133, 40128 Bologna, Italy; renato.iori48@gmail.com; 3 Institute of Organic and Analytical Chemistry (ICOA), Université d’Orléans et the French National Center for Scientific Research (CNRS), Pôle de chimie, rue de Chartres, BP 6759, 45067 Orléans, CEDEX 2, France; patrick.rollin@univ-orleans.fr

**Keywords:** moringin, *Moringa oleifera*, *Staphylococcus aureus* (*S. aureus*), minimum inhibitory concentration (MIC), minimum bactericidal concentration (MBC), time-kill curve, clindamycin-resistant *S. aureus* strain (CC-resistant)

## Abstract

Antimicrobial resistance is one of the major clinical concerns, making the discovery of new antimicrobial drugs desirable. Moringin (MOR), the major isothiocyanate produced from *Moringa oleifera* seeds, could represent an alternative therapeutic strategy to commonly used antibiotics. The aim of our study was to investigate the antimicrobial effect of MOR conjugated with α-cyclodextrin (MOR/α-CD), a complex with an improved solubility and stability in aqueous solutions. Our data demonstrated that MOR/α-CD was able to exert antimicrobial activity against the *S. aureus* reference strains (ATCC 25923, ATCC 6538, and ATCC BAA-977). Moreover, MOR/α-CD showed bacteriostatic effects (MIC = minimum inhibitory concentration = 0.5 mg/mL) and bactericidal properties (MBC = minimum bactericidal concentration = 1 mg/mL) against the overall assessed strains. In addition, MOR/α-CD showed bactericidal activity against the *S. aureus* strain ATCC BAA-977 after treatment with erythromycin (Ery), which induced clindamycin-resistance on the *erm (A)* gene. This evidence led us to assume that MOR/α-CD could be a promising antimicrobial agent against strains with the clindamycin-resistant phenotype (CC-resistant).

## 1. Introduction

*Staphylococcus aureus* (*S. aureus*) is a Gram positive bacteria belonging to the natural microbiota of the healthy people. However, in particular conditions, such as in immunocompromised patients, it can escape the host defenses causing severe infections. In this statement it becomes a serious problem for human health. It is responsible for skin and soft tissue infections and severe human health conditions such as endocarditis, pneumonia, and sepsis [1]. Although clindamycin (CC) stands as one of the most effective antibiotics against *S. aureus* strains [2], the increased emergence of CC resistance during therapy has discouraged some clinicians to prescribe it [2,3]. Natural compounds, such as isothiocyanates (ITCs), may represent a promising alternative strategy to the commonly used antibiotics [4]. It has been demonstrated that ITCs, such as MOR, sulforaphane, and benzyl ITC, show a strong antimicrobial activity [4]. This statement has lead many authors to consider them as promising bactericidal candidates [4]. MOR (4-(α-l-rhamnopyranosyloxy)benzyl ITC) results from myrosinase hydrolysis of glucomoringin (GMG), the most abundant glucosinolate in *Moringa oleifera* seeds [5]. *M. oleifera* seed extracts exert antimicrobial activity against diverse bacteria strains [4]. Furthermore, MOR inhibits *S. aureus* growth, showing bactericidal effect [6,7]. Similarly to most ITCs, MOR is very poorly soluble in water and, in order to overcome this problem, a new complex of MOR with α-CD (MOR/α-CD) was developed [8] and characterized by combining NMR and mass spectrometry experiments [9]. Although the therapeutic potential of MOR/α-CD was recently investigated [8,10], its potential bacteriostatic/bactericidal role has not yet been evaluated. In this study, we investigated whether MOR/α-CD could exert an in vitro antibacterial effect against *S. aureus* reference strains (ATCC 25923, ATCC 6538, and ATCC BAA-977), previously assessed for their sensitivity to MOR. In particular, we evaluated the MOR/α-CD values of MIC and MBC, through time kill curve assays, in order to obtain information about its bacteriostatic and/or bactericidal effect. These results were compared with that obtained using CC, one of the most effective antibiotics against *S. aureus* infections. The comparison with CC was made for all the mentioned reference *S. aureus* strains, sensible to CC. Moreover, since the *S. aureus* resistance to CC could depend on the expression of Ery ribosome methylation (*erm*) genes, we used the ATCC BAA-977 strain, carrying the inducible *erm (A)* gene. To this aim, we induced CC-resistance *erm (A)* gene on the ATCC BAA-977 strain, using a combined CC and Ery treatment [11,12]. After obtaining the resistance, we assessed whether the MOR/α-CD was able to exert its bactericidal effect against the BAA-977 CC-resistant strain.

## 2. Results

### 2.1. Bacterial Strain Growth Curve Determination into the Reference (MHB and CAMHB) Medium

**Figure 1:**
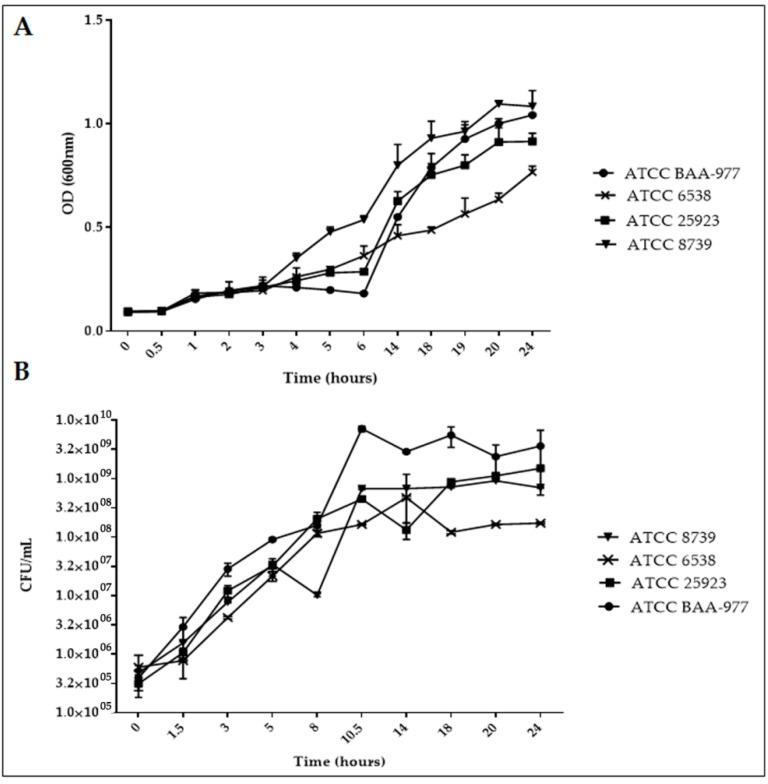
(**A**) The bacterial growth curve (OD/hours). All the strains of *S. aureus* (ATCC BAA-977, ATCC 25923, ATCC 6538) and *E. coli* (ATCC8739) were grown into the reference medium (MHII for the ATCC BAA-977 strain and MHI for the others strains). The starting inoculum was about 5 × 10^5^ CFU/mL. The growth abilities were evaluated by optical density (OD) determination during the time (from time = 0 to time = 24 h). (**B**) Bacterial growth curve (CFU/hours). The same starting inoculum of each strain was used to analyze the unit forming colony (CFU/mL) during the time (from time = 0 to time = 24 h) in the same conditions used for the growth curve determination. Both experiments were done in triplicate to calculate the median and standard deviation by the GraphPad Prism6 software.

The reference strain’s ability to grow into the MIC and MBC standard medium (MHB, Mueller Hinton broth and CAMHB, cation adjusted Mueller-Hinton broth) was evaluated using the same bacterial input (1–7 × 10^5^ CFU/mL) [13]. We assessed the final optical density (OD) and the unit forming colony (CFU) of each strain for up to 24 h of incubation without antibiotics and MOR/α-CD. As indicated in the figure (Figure 1A,B), all the strains were able to grow into the selected medium, although the *S. aureus* ATCC 6538 strain showed the lowest growth ability (OD = 0.76 and CFU/mL = 1.74 × 10^8^) compared to the *S. aureus* ATCC 25923 (OD = 0.91; CFU/mL = 1.5 × 10^9^) and the *S. aureus* ATCC BAA-977 (OD = 1.042 CFU/mL= 3.62 × 10^9^). In addition, despite the fact that *E. coli* ATCC 8739 (OD = 1.083; CFU/mL = 7 × 10^8^) showed a similar OD compared to the BAA-977 strain, its colony count was numerically inferior after 24 h of incubation into the cultural medium.

### 2.2. Determination of the Minimum Inhibitory Concentration for MOR/α-CD

The Minimum Inhibitory Concentration (MIC value) indicates the minimum dose of the antibiotic that determines the visible inhibition of the bacterial growth (lacking turbidity) in the cultural tubes or into the wells of microplates. The objective measure of the bacterial growth was performed reading the bacterial OD spectrophotometrically. We can, therefore, expect that the MIC value represents the dose of the antibiotic that, after 24 h of incubation, determined similar OD reached by the input bacteria at the time = 0 h (not growth) or the OD reached by the positive control (the antibiotic that exerts known antimicrobial activity against the assessed strains). In this experiment, the MIC value of the MOR/α-CD was performed according to the universal protocol indicated by EUCAST guidelines [13].

**Figure 2:**
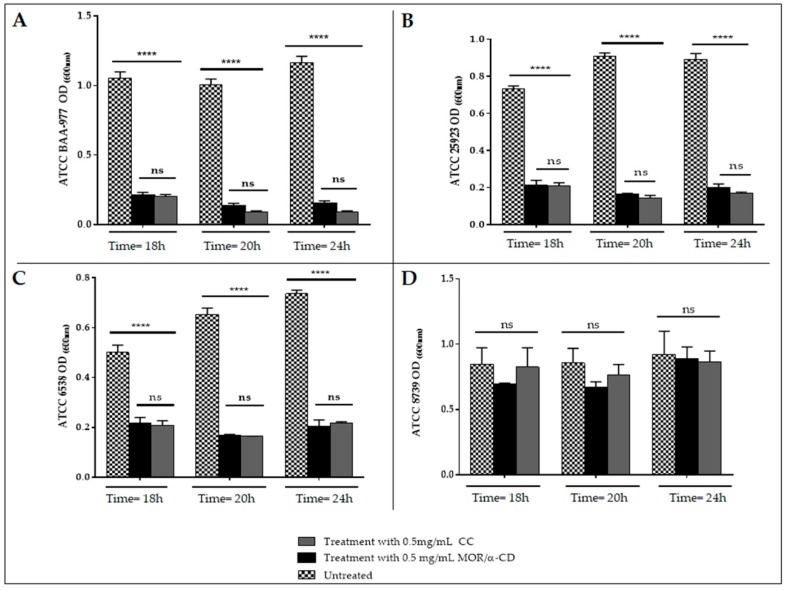
The MIC evaluation of MOR/α-CD against *S. aureus* ATCC BAA-977 (**A**), *S. aureus* ATCC 25923 (**B**), *S. aureus* ATCC 6538 (**C**) and *E. coli* ATCC 8739 (**D**); MIC value of MOR/α-CD was evaluated using a starting inoculum of 5 × 10^5^ CFU/mL and measuring the optical density (OD) at 18 h–20 h and 24 h. We assessed different doses of MOR/α-CD, but in the graph, we represented only the dose of 0.5 mg/mL, which was able to affect the growth after 24 h of incubation. Experiments were done in triplicate to obtain the mean and the standard deviation (SD). Statistical analysis (Two-way ANOVA) was performed by the GraphPad Prism6 software and the *p* value < 0.0001 (****) showed significant difference.

The results indicated that a dose of 0.5 mg/mL of MOR/α-CD was able to counteract the *S. aureus* BAA-977 growth after 24 h of treatment (Figure 2A). Especially the treatment with 0.5 mg/mL did not exert significant different with that observed using the same CC antibiotic concentration. Moreover, after 24 h of the treatment, the bacterial OD reading was similar to that observed for the bacterial input (time = 0) and the positive control (the treatment with CC) (Figure S1). We indicated that 0.5 mg/mL was the minimum inhibitory concentration (MIC value) of the MOR/α-CD able to inhibit the growth of *S. aureus* BAA-977. Indeed, the treatment with lower concentration (from 0.25 mg/mL to 0.002 mg/mL) did not exert the same result after 24 h of incubation (Figure S1).

The MIC value of MOR/α-CD against *S. aureus* ATCC 25923 and ATCC 6538 was also 0.5 mg/mL after 24 h of treatment (Figure 2B,C), although the best MOR/α-CD antimicrobial activity appeared after 20 h of treatment with MIC value of 0.25 mg/mL (Figures S2 and S3), probably due to the lower number of bacteria.

*E. coli* represented the negative control. Indeed, after 24 h of incubation, no growth inhibition was observed by testing CC or MOR/α-CD against *E. coli* ATCC 8739 using a dose of 0.5 mg/mL of both CC or MOR/α-CD (Figure 2D). Although a dose of 0.25 mg/mL of MOR/α-CD showed a slight decrease of the *E. coli* growth (OD) compared to the ineffective CC antibiotic (Figure S4), this result did not suggest a good antimicrobial activity.

**Figure 3:**
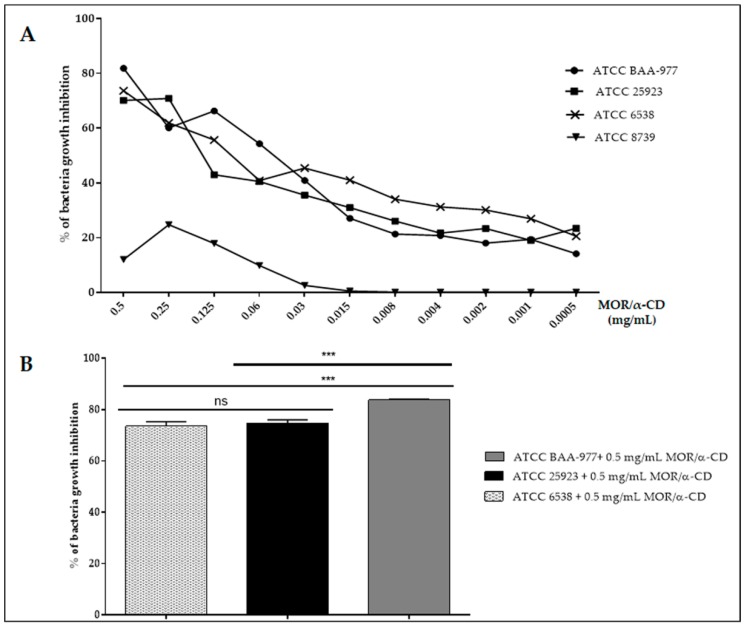
The percentage of relative bacterial growth inhibition among the strains. (**A**) Percentage of bacterial growth inhibition by different doses of MOR/α-CD complex calculated as [(Ac − At)/Ac] × 100, where Ac was the OD reached by untreated bacteria after 24 h of incubation and At was the OD reached by treated bacteria after 24 h of incubation. (**B**) A statistical analysis (one-way ANOVA test) was performed by GraphPad Prism6 software to compare the most effective dose of the MOR/α-CD complex (0.5 mg/mL) among the strains. Experiments were assessed in triplicate to obtain the mean and the standard deviation (SD). *p* value < 0.0002 (***) showed a significant difference.

In order to compare the MOR/α-CD antimicrobial activity among the assessed strains, we analyzed the percentage (%) of the bacterial growth inhibition calculated as [(Ac − At)/Ac] × 100, where Ac was an average of three replicates of light absorption values at wavelength 600 nm of the negative controls (the OD reached by untreated bacteria after 24 h of incubation) and At was an average of the three replicates of light absorption values at a wavelength of 600 nm of the samples (the OD reached by treating bacteria after 24 h of incubation with different doses of the MOR/α-CD). The results indicated that, after 24 of treatment, the sensible strains (ATCC 25923, ATCC 6538 and ATCC BAA-977) were similarly affected by the bacterial growth ability (from to 70% up to about 82% of growth inhibition) although MOR/α-CD appeared to inhibit 10% more of ATCC BAA-977 growth than the other strains (Figure 3A,B) notwithstanding the fact that ATCC BAA-977 showed faster growth and a higher number of CFU (Figure 1A,B). The remaining 25% of the bacterial growth inhibition at a lower dose of MOR/α-CD (up to 0.005%) could be referred to as the percentage of the quiescent bacteria that restarted the exponential growth phase.

### 2.3. Determination of Minimum Bactericidal Concentration for MOR/α-CD

**Figure 4:**
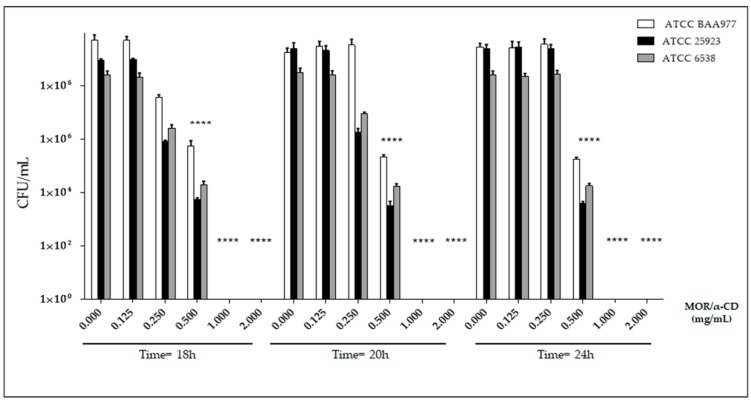
The time kill curve assay. MOR/α-CD bactericidal activity was assessed against *S. aureus* ATCC BAA-977, ATCC 25923, and ATCC 6538 strains using the MIC dose (0.5 mg/mL) of MOR/α-CD and from 2 up to 4-fold the value of MIC for each strain (0.5, 1 and 2 mg/mL). The colony count was made after 18 h, 20 h, and 24 h of treatment. Experiments were assessed in triplicate to obtain the mean and the standard deviation (SD). A statistical analysis (Two way ANOVA) was performed by the GraphPad Prism6 software and the *p* value < 0.0001 (****) showed a significant difference between the dose of 0.5 mg/mL, 1 mg/mL, and 2 mg/mL of MOR/α-CD and the untreated bacteria (0 mg/mL) for each strain at different time points.

The MBC value is defined as the antimicrobial concentration that ensures the killing of 99.9% of the bacterial input (1–7 × 10^5^ CFU/mL). Indeed, we could expect a ≥ 3log_10_ decreasing of the viable bacteria (CFU/mL) compared to the number of viable bacteria used to starting the experiment at the time = 0 h (the bacterial input) [14]. The MBC value can be determined by time-kill curves. This method is similar to the one used for the MIC determination but, following the incubation with MOR/α-CD, an aliquot of bacteria was seeded into the reference plate to observe the number of the viable bacteria after the treatment. In addition, as indicated in the standard protocol [14], we used not only the amount of MOR/α-CD corresponding to the MIC value but also from two, four and multiple concentrations of the MIC. The rationale is that the MIC concentration could exert only bacteriostatic effect given the same colony count compared to the bacterial input. The use of two, four, and multiple MOR/α-CD concentrations could allow to observe bactericidal effect. Results indicated that 1 mg/mL of MOR/α-CD represented the minimum bactericidal concentration (MBC) value for each strain. Indeed, no viable bacteria (CFU/mL) have been observed after the treatment with this dose of MOR/α-CD (Figure 4). The same result was observed assessing a dose of 2 mg/mL of MOR/α-CD (Figure 4). Interestingly, the dose of 0.5 mg/mL of MOR/α-CD that represents the MIC value (or the MOR/α-CD concentration that did not show visible bacterial growth measured by OD reading) exerted only a bacteriostatic effect. Indeed, 0.5 mg/mL of MOR/α-CD kept the same number of the *S. aureus* ATCC BAA-977 input used for the time = 0 h and only a 1-log10 reduction of the bacterial input for the other strains assessed (Figure 4).

### 2.4. MOR/α-CD against BAA-977 Strain with a CC-Resistance Phenotype

**Figure 5:**
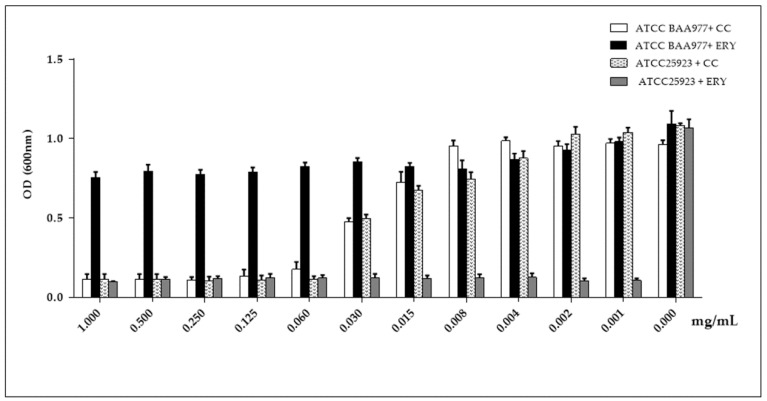
The Ery MIC evaluation against ATCC BAA-977 and ATTC 25923. A standard bacterial inoculum (5 × 10^5^ CFU/mL) was used and the OD reached was read after 24 h of treatment. The result was compared to the MIC of the CC antibiotic. The experiments were assessed in triplicate in order to obtain the mean and the standard deviation (SD).

**Figure 6:**
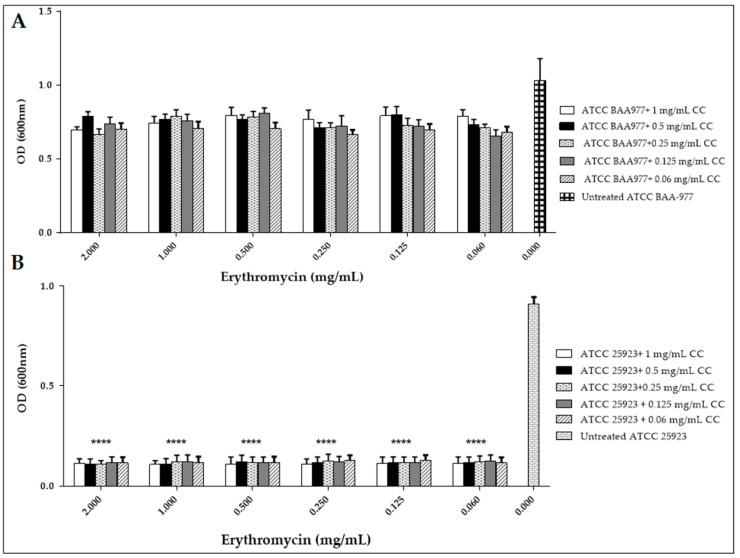
(**A**) The antibiotic combined assay to induce CC-resistance in the BAA-977, carrying the *erm (A)* inducible CC-resistance gene. The experiment was performed using the doses of CC that naturally exerted antimicrobial activity (1, 0.5, 0.25, 0.125, 0.06 mg/mL) combined with different doses of Ery (from 2 mg/mL to 0.06 mg/mL) against BAA-977. (**B**) The same experiment was done using the ATCC 25923 strain, lacking the resistance inducible gene. A statistical analysis (Two way ANOVA) was performed by the GraphPad Prism6 software and the *p* value < 0.0001 (****) showed a significant difference between the treatment with 1 mg/mL of MOR/α-CD of the resistant strain in each combined well and the untreated bacteria (0 mg/mL).

Finally, we investigated the antimicrobial activity of the MOR/α-CD against bacteria with the CC-resistance phenotype. To this aim, we performed a protocol known to induce the CC resistance phenotype on the bacteria carrying the *erm* genes [11,12]. The protocol includes a combined treatment with CC and Ery. The experiment was done using the ATCC BAA-977 strain carrying the *erm (A)* gene. We first evaluated the antimicrobial activity of different doses of Ery and CC alone against the ATCC BAA-977 (Figure 5) compared to the same treatment against the ATCC 25923 strain lacking the *erm* genes (Figure 5). Then, we assessed the combined Ery/CC treatment on the BAA-977 strain in order to induce the *erm* A gene obtaining the CC-resistant strain (Figure 6A and Figure S5). To perform the combined treatment, each different dose of CC (from 1 to 0.006 mg/mL), exerting a growth inhibitory activity was assessed with every one dose of Ery (from 2 to 0.002 mg/mL) to have a better combination that ensures the expression of the CC resistant phenotype. The same combined treatment was carried out against the ATCC 25923 to demonstrate that the treatment on the strain lacking the *erm* genes did not confer the CC resistance (Figure 6B and Figure S6).

**Figure 7:**
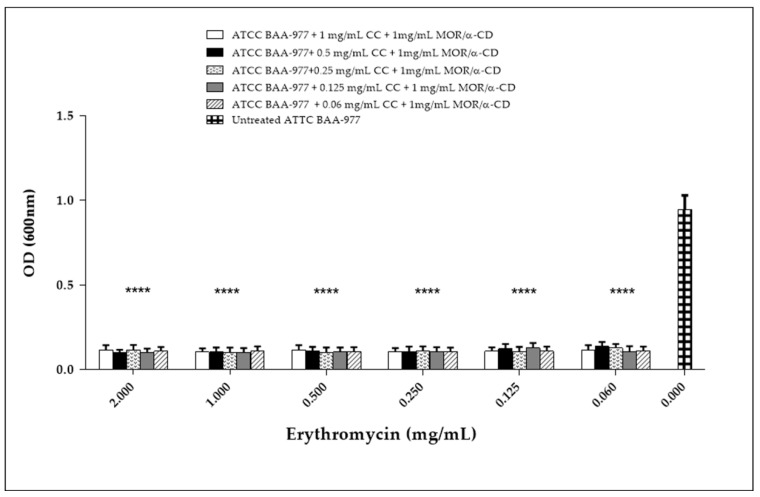
The MOR/α-CD inhibition activity against the ATCC BAA-977 CC-resistant strain. The experiment was carried out adding 1 mg/mL of MOR/α-CD for each microplate well filled with the combined doses of CC (1, 0.5, 0.25, 0.125, 0.06 mg/mL) and Ery (from 2 mg/mL to 0.002 mg/mL). The MOR/α-CD inhibition activity was evaluated by measuring the OD reached after 24 h of treatment. The experiments were assessed in triplicate to obtain the mean and the standard deviation (SD). A statistical analysis (Two way ANOVA) was performed by the GraphPad Prism6 software and the *p* value < 0.0001 (****) showed a significant difference between the treatment with 1 mg/mL of MOR/α-CD of the resistant strain in each combined well and the untreated bacteria (0 mg/mL).

In the wells with Ery alone, the BAA-977 strain displayed a normal growth, suggesting its natural resistance to Ery. Indeed, the Ery treatment did not exert any growth inhibition (Figure 5). To the contrary, it strongly affected the growth of *S. aureus* ATCC 25923 used as the test control (Figure 5). Moreover, in the wells with the combination of CC and Ery antibiotics, also in the presence of an inhibitory concentration of CC, the BAA-977 strain was able to grow thanks to the induction of the bacterial *erm (A)* gene by the Ery treatment (Figure 6A and Figure S5). Each Ery/CC combination exerted a similar effect. Only the combination of 1, 0.5, or 0.25 of CC with 0.002 of Ery was shown to affect the bacterial growth. This result was probably due to the high doses of CC and contemporary to the low dose of Ery, which was not able to determine gene induction (Figure 6A and Figure S5). No growth was observed by testing the ATCC 25923 strain in the same conditions (Figure 6B and Figure S6). In order to evaluate the antimicrobial activity of MOR/α-CD against the CC-resistant BAA-977 strain, we performed a similar combined assay, adding 1 mg/mL of MOR/α-CD on each well of the microplates with the Ery/CC combinations. As expected, in the presence of MOR/α-CD, no bacterial growth was observed (Figure 7 and Figure S7). Moreover, no CFU was counted by seeding 10 μL from each antibiotic combined well, after 24 h of MOR/α-CD treatment (data not shown).

## 3. Discussion

The request for new antibiotics has lead the scientific community to investigate the antimicrobial properties of plant-derived products and their potential use as antimicrobial agents.

*M. oleifera* is one of the most investigated plants, as suggested by a number of studies which have demonstrated that whole extracts from the different parts (seeds, bark, leaves, or root) of the plant were able to counteract the bacterial growth.

Among plant-derived compounds, ITCs have been widely utilized for food preservation and plant pathogen control, thanks to their anti-microbial activity [15], but still little is known of their antibacterial activity in the human health context.

Besides, ITCs isolated from *M*. *oleifera*, such as MOR (4-(α-l-rhamnopyranosyloxy) benzyl ITC), have been scarcely studied for their antimicrobial properties.

Our study has focused on the potential antimicrobial properties of a new complex formed by MOR and α-CD (MOR/α-CD). MOR is an ITC isolated from *M. oleifera* seeds known to exert an antibacterial effect against *S. aureus* strains. α-CD is a cyclic hexamer of d-glucose that improves the solubility of MOR [16]. Although the effects of MOR/α-CD on human health was investigated, its antimicrobial activity has not been assessed to date. In order to investigate the antimicrobial properties of MOR/α-CD in a more in-depth manner, we focused on the MIC and the MBC evaluation, comparing these results to those obtained using CC, one of the most effective antibiotics against *S. aureus* infection. Our results demonstrated that a dose of 0.5 mg/mL (MIC) of MOR/α-CD determined the inhibition of the growth of all *S. aureus* strains included in the study (*S. aureus* ATCC 25923, ATCC 6538 and ATCC BAA-977), according to that previously described by our research group. Indeed, our previous study reported that a dose of 1.52 mg/mL of MOR was able to counteract the growth of the *S. aureus* BAA-977 strain (inhibition halo of about 25 ± 1 mm), suggesting the effectiveness of the MOR activity against *S. aureus* strains [7]. This result was improved by assessing the MIC value through a dose dependent broth microdilution assay, which led us to indicate that 0.5 mg/mL of MOR/α-CD as sufficient to counteract the *S. aureus* BAA-977 growth. Our results were also in agreement with those of Padla et al. [6] and Peixoto et al. [17]. The latter demonstrated that *M. oleifera* showed strong antimicrobial properties against Gram positive bacteria including the *S. aureus* ATCC 25923 strain and Padla et al. demonstrated that a dose of 10 mg/mL of MOR showed results similar to those of ofloxacin against the *S. aureus* ATCC 6538 strain. In addition, as demonstrated by the above authors, neither the total extract nor the isolated MOR affected the growth of *E. coli* strains. According to that, we used the *E. coli* strain as a negative control demonstrating that 0.5 mg/mL of MOR/α-CD was not sufficient to counteract the *E. coli* strain.

To determine whether MOR/α-CD exhibited a bactericidal or bacteriostatic effect, we performed a time-kill curve. The experiment was performed using different doses of MOR/α-CD according to the standard protocol. We used 0.5 mg/mL of MOR/α-CD, which represents its MIC value against all the reference strains included in the study, and 2-fold (1 mg/mL) and 4-fold (4 mg/mL) the MIC value. Interestingly, the dose of 0.5 mg/mL determined only a bacteriostatic effect maintaining the same number of the bacteria input. When a dose of 1 mg/mL was used, no bacterial CFU was observed, indicating that 1 mg/mL of the MOR/α-CD exerted a bactericidal effect. These results are in agreement with those of Padla et al. suggesting the bactericidal properties of MOR [6]. The comparison between MOR/α-CD and the CC antibiotic suggested that CC determined an inhibitory effect at a lower dose (MIC = 0.06 mg/mL) than MOR/α-CD (MIC = 0.5 mg/mL), showing better antimicrobial properties against the strains included in the study. Despite this outcome, it is important to underline that CC is one of the most effective antibiotics against *S. aureus* infections, including infection sustained by MRSA strains [18]. Furthermore, CC is able to counteract a high bacterial burden at the infection site [2]. Interestingly, comparing the ability of 1 mg/mL of MOR/α-CD to reduce the bacterial input to the same dose of CC, MOR/α-CD exerts a bactericidal effect while CC showed only bacteriostatic properties. In addition, MOR/α-CD could be useful against strains with CC-resistant phenotype [3]. Concerning this topic, we evaluated whether MOR/α-CD bactericidal effects were maintained against the BAA-977 after the induction of CC-resistance. Indeed, the BAA-977 strain carries an inducible *erm (A)* gene that can confer the resistance to the CC antibiotic after the induction by Ery treatment [11,12]. To this end, we performed a combined assay for each microplate well using different doses of CC that exert bacteriostatic effects and different doses of Ery. We also evaluated the effect of a treatment with Ery alone. The evaluation of the effectiveness of Ery alone against the BAA-977 strain suggested that the BAA-977 was not affected by Ery treatment. When the BAA-977 was treated with a combination of Ery and CC, the antibiotic CC lost its bacteriostatic properties and the growth of the BAA-977 strain could be observed. The experiment demonstrated that the Ery treatment induced the resistance to CC in the BAA-977 strain. Once the resistant strain was obtained, we assessed if 1 mg/mL of MOR/α-CD could maintain a bactericidal effect against the CC-resistant strain. Surprisingly, our results demonstrated that the treatment with 1 mg/mL of MOR/α-CD determined no bacterial growth (OD) and also no bacterial CFU on the plate. This is a very interesting result, indeed the same dose of MOR/α-CD that exerts a bactericidal effect against the sensible strain was also effective against the CC-resistant strain, suggesting a possible synergic use of MOR/α-CD with the CC antibiotic or in its use alone. Moreover, MOR/α-CD is not affected by the presence of the *erm (A)* gene, suggesting that this molecular mechanism underlying the CC-resistance was not involved in the MOR/α-CD effectiveness.

## 4. Materials and Methods

### 4.1. Isolation of MOR

MOR was produced via myrosinase-catalyzed hydrolysis of GMG isolated from *Moringa oleifera* (fam. *Moringaceae*) seeds (cake powder PKM2 provided by Indena India Pvt. Ltd.; Bangalore, India) at the Bologna laboratory (CREA-AA; previously CIN), and was purified by reverse-phase chromatography, according to the procedure described by Brunelli et al. [19]. Its identity was confirmed by NMR spectrometry, and the purity has been estimated >99% as reported by Muller et al. [5]. The soluble complex MOR/α-CD was obtained by adding 103 mg of solid MOR to a solution of 300 mg α-CD (Wacker Chemie AG, München, Germany) in 3.0 mL of water, with a 1:1 M ratio of the two constituents [9]. The resulting aqueous solution was filtered with a 0.45 μm filter, then freeze-dried (Edwards model DO1; Milan, Italy) [8]. One gram of the complex contained 242.45 mg MOR. Characterization of the MOR/α-CD inclusion complex was recently published by Mathiron et al. [9].

### 4.2. Antibiotics and MOR/α-CD Dilution

CC (Clindamycin hydrochloride, a lincosamide antibiotic) was purchased from Sigma Aldrich (Saint Louis, MI, USA) and used accordingly as indicated by EUCAST guidelines (from 0.5 mg/mL to 0.001 mg/mL). Ery was purchased from Sigma Aldrich (Saint Louis, MI, USA). To perform the MIC, we used 1 to 0.001 mg/mL of CC and 2 to 0.002 mg/mL of Ery. To perform the combined antibiotic assay, we used all combinations between 1–0.5–0.25–0.125–0.06 mg/mL of CC and 2–1–0.5–0.25–0.125–0.06–0.03–0.015–0.008–0.004–0.002 mg/mL of Ery. We dissolved the CC in PBS to obtain a concentration of 50 mg/mL and then it was diluted in the same medium used for the MIC and MBC determinationz. We dissolved CC in Mueller-Hinton broth (MHB) for the tests against ATCC 25923, ATCC 6538, ATCC 8739, and the cation-adjusted Mueller-Hinton broth (CAMHB) for the test against ATCC BAA-977 strain. Concerning the Ery, we resuspended the powder in ethanol to obtain a concentration of 50 mg/mL and then we performed the dilutions in Mueller-Hinton broth (MHB) for the test against ATCC 25923, and the cation-adjusted Mueller-Hinton broth (CAMHB) for the test against ATCC BAA-977 strain. Since 1 g of MOR/α-CD contains 242.45 mg of MOR, we calculated the useful concentration of MOR/α-CD that included the suitable amount of active MOR (from 2 mg/mL to 0.001 mg/mL) in MOR/α-CD without the inert contribution of α-CD. We used 0.5 mg/mL and a two-fold dilution of MOR/α-CD to perform MIC assay, 2 mg/mL and a two-fold dilution of the complex to performed time-kill curve assay and 1 mg/mL to the combined assay. We dissolved the MOR/α-CD powder in the same medium used for carrying out the experiments as described for the antibiotics.

### 4.3. S. aureus Strains and Growth Curve

The microbial strains used in the study were obtained from an American type cell collection (ATCC) and included *S. aureus* ATCC 6538, *S. aureus* ATCC 25923, *S. aureus* ATCC BAA-977, and *E. coli* ATCC 8739. *E. coli* ATCC 8739 represented the negative control [6]. The lyophilized strains were cultured in the appropriate medium and then isolated in the useful agar plates, according to the manufacture instructions. MIC and MBC evaluations were carried out using cation-adjusted Mueller-Hinton broth (CAMHB) and agar (CAMHA) (Difco) and Mueller-Hinton broth (MHB) and agar (MHA) (Difco). The strains ability to grow in CAMHB and MBH were evaluated using a started bacterial inoculum of 1–7 × 10^5^ CFU/mL and the reached turbidity (optical density = OD) was analyzed at 600 nm during 24 h of growth in agitation at 37 °C. The experiments were performed in 96-well microplates and the VICTOR Nivo Multimode Microplate Reader (PerkinElmer, Waltham, MA, USA), equipped with an optical filter of 600 nm, was used to measure the bacterial turbidity indicating bacteria growth during the time. Simultaneously, aliquots (20 μL) from every well were collected to evaluate the number of CFU at the same time points. The bacterial input was evaluated by count on the suitable agar plates. Ten-fold dilutions were performed to obtain the suitable amount of colony in the plate. The experiments were done in triplicate to perform a statistical analysis.

### 4.4. MOR/α-CD Inhibitory Concentration (MIC)

The MIC values of MOR/α-CD against *S. aureus* strains were determined by the broth microdilution method in sterile 96 well plates, according to EUCAST guidelines [13]. To prepare the bacterial inoculum, we used the “broth culture method” [13]; from plates less than 30 h old, the colony is touched with a loop and then transferred to CAMHB (ATCC BAA-977) and MHB (for the others strains). The cultures were than incubated at 35 ± 2 °C up to the growth reached a turbidity equal to or greater than 0.5 McFarland standard. The culture is adjusted with broth to give a turbidity equivalent to the standard of 0.5 McFarland corresponding to 1.8 × 10^8^ CFU/mL. This value was measured by a photometrical method and by agar colony count. This bacteria concentration was diluted in broth until the final bacterial density was 1–7 × 10^5^ CFU/mL, representing the input of bacteria used for the MIC evaluation. Concerning MOR/α-CD, serial two fold dilutions (from 0.5 mg/mL to 0.001 mg/mL) for each well were performed to assess the MIC value for MOR/α-CD, that is, a MOR/α-CD minimal concentration that inhibits the *S. aureus* growth during 24 h of incubation at 37 °C in agitation. These results were compared to the antibacterial activity of the CC antibiotic used at the same concentrations. The results were obtained reading the bacterial turbidity (optical density OD) during the time up to 24 h (reading at time = 0 h, Time = 18 h, Time = 20 h, and Time = 24 h) by the VICTOR Nivo Multimode Microplate Reader (PerkinElmer) equipped with an optical filter of 600 nm. The experiments were done in triplicate to perform a statistical analysis.

### 4.5. MOR/α-CD Minimum Bactericidal Concentration (MBC)

The time-kill curve method is done by a microdilution assay using an amount of MOR/α-CD corresponding to the MIC value and from 2 up to 4-fold the value of the MIC for each strain (from 2 mg/mL to 0.5 mg/mL). The protocol used was referred to from the standard guidelines [14] and performed in sterile 96 well plates with flat bottoms, each containing a final volume of 0.2 mL of culture with or without antibiotics. We assessed 2 to 0.125 mg/mL of MOR/α-CD and the same inoculum for the MIC determination. During the treatment, 20 μL from each well were collected at different time points (18, 20, and 24 h of incubation) followed by bacterial dilution and seeding in the reference plates (MHA and MHA II). Finally, the colonies were counted, and the results were represented in a graph with the survivor colony count on the ordinate in the logarithmic scale and the time on the abscissa on the arithmetic scale. The experiments were done in triplicate to perform a statistical analysis.

### 4.6. MOR/α-CD Inhibitory (MIC) and Bactericidal Concentration (MBC) against S. aureus ATCC BAA-977 with CC-Resistant Phenotype Induced by Ery Treatment

To induce the CC-resistance in the *S. aureus* ATCC BAA-977 strain carrying the inducible CC-resistance *erm* (*A)* gene, we used a combination of different doses of CC and Ery for each well of a microplate. We used a combination of 1–0.5–0.25–0.125–0.06 mg/mL of CC with 2–1–0.5–0.25–0.125–0.06–0.003–0.015–0.08–0.004–0.02 mg/mL of Ery for each well. The reached OD was evaluated during the time (from 18 to 24 h of incubation) compared to that observed when using CC or Ery alone. Similar experiment has been performed using the ATCC 25923 strain lacking for *erm* genes and used as a positive control for Ery treatment. We carried out the experiments testing the same inoculum used for the MIC determination. To assess the MOR/α-CD’s ability to inhibit the growth of the resistant strain, we performed a similar experiment adding 1 mg/mL of MOR/α-CD for each combined well and reading the reached OD at different time points (18 h, 20 h, and 24 h). The colony count at the same time point was also evaluated by seeding 10 μL from each well. The experiments were done in triplicate to perform a statistical analysis.

### 4.7. Statistical Analysis

A statistical analysis was performed by the GraphPad Prism6 software using one way and two way ANOVA followed by Bonferroni post-hoc test. A *p* value < 0.05 was considered statistically significant. The results were shown as mean ± SD.

## 5. Conclusions

The antimicrobial activity of MOR, together with its acquired solubility, suggested that MOR/α-CD may represent a promising antimicrobial candidate. Our results indicated that MOR/α-CD showed bacteriostatic activity at a dose of 0.5 mg/mL (the MIC value) against *S. aureus* reference strains although its antimicrobial activity was lower if compared to the CC antibiotic (MIC = 0.06 mg/mL), one of the most effective antibiotics against the *S. aureus* infection. Moreover, a dose of 1 mg/mL (the MBC value) of MOR/α-CD is able to exert bactericidal activity against the BAA-977 wild type (CC-sensible) and against the BAA-977 strain, when its CC-inducible resistance *erm (A)* gene was induced by the Ery treatment, suggesting its potential use against the CC-resistant strains. This study encourages us to further investigate the potential use of MOR/α-CD as an antimicrobial agent.

## Supplementary Materials

The following are available online. Figure S1: MIC evaluation of MOR/α-CD against *S. aureus* ATCC BAA-977. Figure S2: MIC evaluation of MOR/α-CD against *S. aureus* ATCC 25923. Figure S3: MIC evaluation of MOR/α-CD against *S. aureus* ATCC 6538. Figure S4: MIC evaluation of MOR/α-CD against *E. coli* ATCC 8739. Figure S5: Antibiotic combined assay to induce CC-resistance in the ATCC BAA-977 strain, carrying the *erm* (*A*) inducible CC-resistance gene. Figure S6: Antibiotic combined assay against the ATCC 25923 lacking of resistance inducible gene. Figure S7: MOR/α-CD growth inhibition activity against the ATCC BAA-977 CC-resistant strain.

## References

[1] Bottega A., Rodrigues M.D., Carvalho F.A., Wagner T.F., Leal I.A.S., Dos Santos S.O., Rampelotto R.F., Horner R. (2014). Evaluation of constitutive and inducible resistance to clindamycin in clinical samples of *Staphylococcus aureus* from a tertiary hospital. Rev. Soc. Bras. Med. Trop..

[2] Lewis J.S., Jorgensen J.H. (2005). Inducible clindamycin resistance in staphylococci: Should clinicians and microbiologists be concerned?. Clin. Infect. Dis..

[3] Patel M., Waites K.B., Moser S.A., Cloud G.A., Hoesley C.J. (2006). Prevalence of inducible clindamycin resistance among community- and hospital-associated *Staphylococcus aureus* isolates. J. Clin. Microbiol..

[4] Romeo L., Iori R., Rollin P., Bramanti P., Mazzon E. (2018). Isothiocyanates: An overview of their antimicrobial activity against human infections. Molecules.

[5] Muller C., van Loon J., Ruschioni S., De Nicola G.R., Olsen C.E., Iori R., Agerbirk N. (2015). Taste detection of the non-volatile isothiocyanate moringin results in deterrence to glucosinolate-adapted insect larvae. Phytochemistry.

[6] Padla E.P., Solis L.T., Levida R.M., Shen C.C., Ragasa C.Y. (2012). Antimicrobial isothiocyanates from the seeds of moringa oleifera lam. Z. Naturforsch. C.

[7] Galuppo M., De Nicola G.R., Iori R., Dell’Utri P., Bramanti P., Mazzon E. (2013). Antibacterial activity of glucomoringin bioactivated with myrosinase against two important pathogens affecting the health of long-term patients in hospitals. Molecules.

[8] Giacoppo S., Iori R., Rollin P., Bramanti P., Mazzon E. (2017). Moringa isothiocyanate complexed with alpha-cyclodextrin: A new perspective in neuroblastoma treatment. BMC Complement. Altern. Med..

[9] Mathiron D., Iori R., Pilard S., Soundra Thangavelu R., Landy D., Mazzon E., Rollin P., Djedaïni-Pilard F. (2018). A combined approach of nmr and mass spectrometry techniques applied to the alpha-cyclodextrin/moringin complex for a novel bioactive formulation. Molecules.

[10] Giacoppo S., Rajan T.S., Iori R., Rollin P., Bramanti P., Mazzon E. (2017). The a-cyclodextrin complex of the moringa isothiocyanate suppresses lipopolysaccharide-induced inflammation in raw 264.7 macrophage cells through akt and p38 inhibition. Inflamm. Res..

[11] Steward C.D., Raney P.M., Morrell A.K., Williams P.P., McDougal L.K., Jevitt L., McGowan J.E., Tenover F.C. (2005). Testing for induction of clindamycin resistance in erythromycin-resistant isolates of *Staphylococcus aureus*. J. Clin. Microbiol..

[12] Matsuoka M., Inoue M., Nakajima Y., Endo Y. (2002). New *erm* gene in *Staphylococcus aureus* clinical isolates. Antimicrob. Agents Chemother..

[13] European Committee for Antimicrobial Susceptibility Testing (EUCAST) of the European Society of Clinical Microbiology and Infectious Diseases (ESCMID) (2003). Determination of minimum inhibitory concentrations (mics) of antibacterial agents by broth dilution. Clin. Microbiol. Infect..

[14] Barry A.L., Craig W.A., Nadler H., Reller L.B., Sanders C.C., Swenson J.M. (1999). Methods for Determining Bactericidal Activity of Antimicrobial Agents: Approved Guideline.

[15] Delaquis P.J., Mazza G. (1995). Antimicrobial properties of isothiocyanates in food preservation. Food Technol..

[16] Roselli C., Perly B., Rollin P. (2004). Complexes for Immobilizing Isothiocyanate Natural Precursors in Cyclodextrins, Preparation and Use. US Patent.

[17] Peixoto J.R.O., Silva G.C., Costa R.A., Fontenelle J.L.D., Vieira G.H.F., Fonteles A.A.F., Vieira R.H.S.D.F. (2011). In vitro antibacterial effect of aqueous and ethanolic moringa leaf extracts. Asian Pac. J. Trop. Med..

[18] Klevens R.M., Morrison M.A., Nadle J., Petit S., Gershman K., Ray S., Harrison L.H., Lynfield R., Dumyati G., Townes J.M. (2007). Invasive methicillin-resistant *Staphylococcus aureus* infections in the united states. J. Am. Med. Assoc..

[19] Brunelli D., Tavecchio M., Falcioni C., Frapolli R., Erba E., Iori R., Rollin P., Barillari J., Manzotti C., Morazzoni P. (2010). The isothiocyanate produced from glucomoringin inhibits nf-kb and reduces myeloma growth in nude mice in vivo. Biochem. Pharmacol..

