# Supplementary material for: The α-Cyclodextrin/Moringin Complex: A New Promising Antimicrobial Agent against Staphylococcus aureus
Source: Molecules. 2018 Aug 21;23(9):2097. doi: 10.3390/molecules23092097 (PMC6225138; doi:10.3390/molecules23092097)
Supplement: Supplementary file 1 [file molecules-23-02097-s001.pdf]

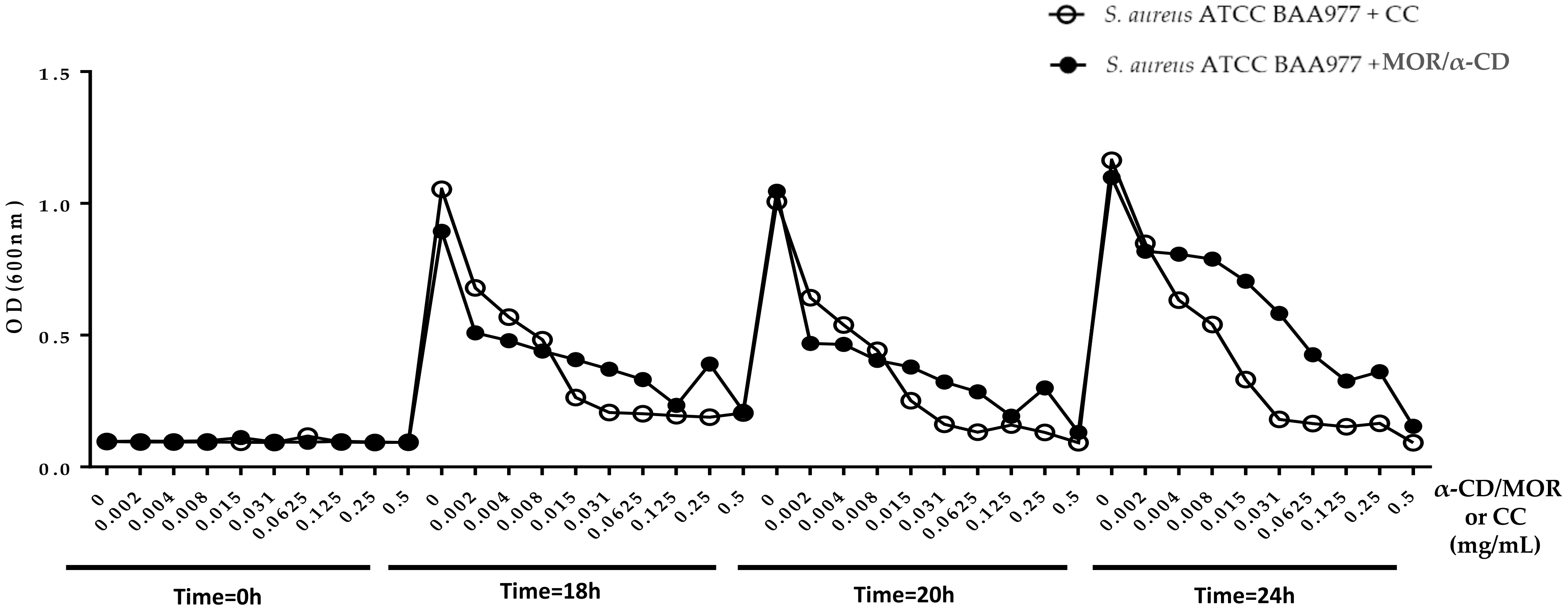

S1: MIC evaluation of MOR/α-CD against *S. aureus* ATCC BAA-977. The MIC value of MOR/α-CD was evaluated using a bacterial input of $5x10^5$ CFU/mL and measuring the optical density (OD) at time=0, time=18h, time=20h and time=24h. We assessed two fold dilution of MOR/α-CD (from 0,5 mg/mL to 0,002 mg/mL) compared to the same dilutions of clindamycin. The untreated bacteria (0 mg/mL of MOR/α-CD and 0 mg/mL of the clindamycin) represent the negative control.

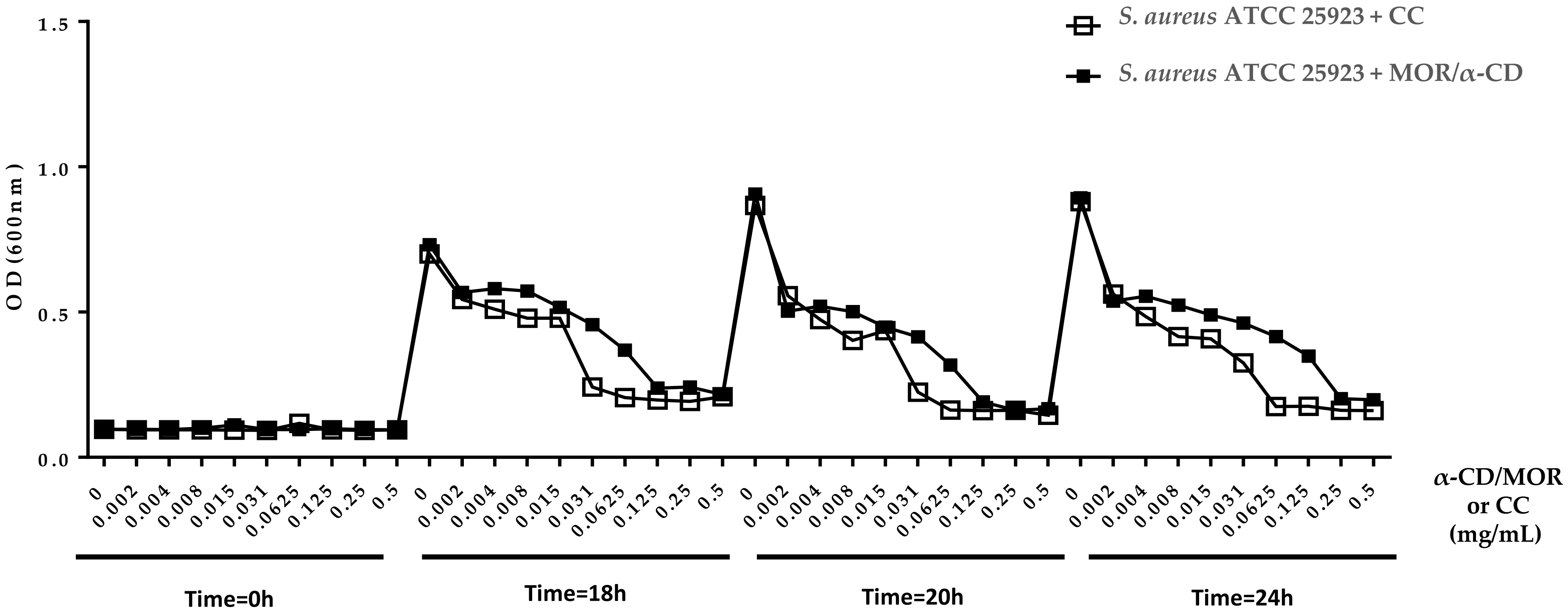

S2: MIC evaluation of MOR/α-CD against *S. aureus* ATCC 25923. The MIC value of MOR/α-CD was evaluated using a bacterial input of $5x10^5$ CFU/mL and measuring the optical density (OD) at time=0, time=18h, time=20h and time=24h. We assessed two fold dilution of MOR/α-CD (from 0,5 mg/mL to 0,002 mg/mL) compared to the same dilutions of clindamycin. The untreated bacteria (0 mg/mL of MOR/α-CD and 0 mg/mL of the clindamycin) represent the negative control.

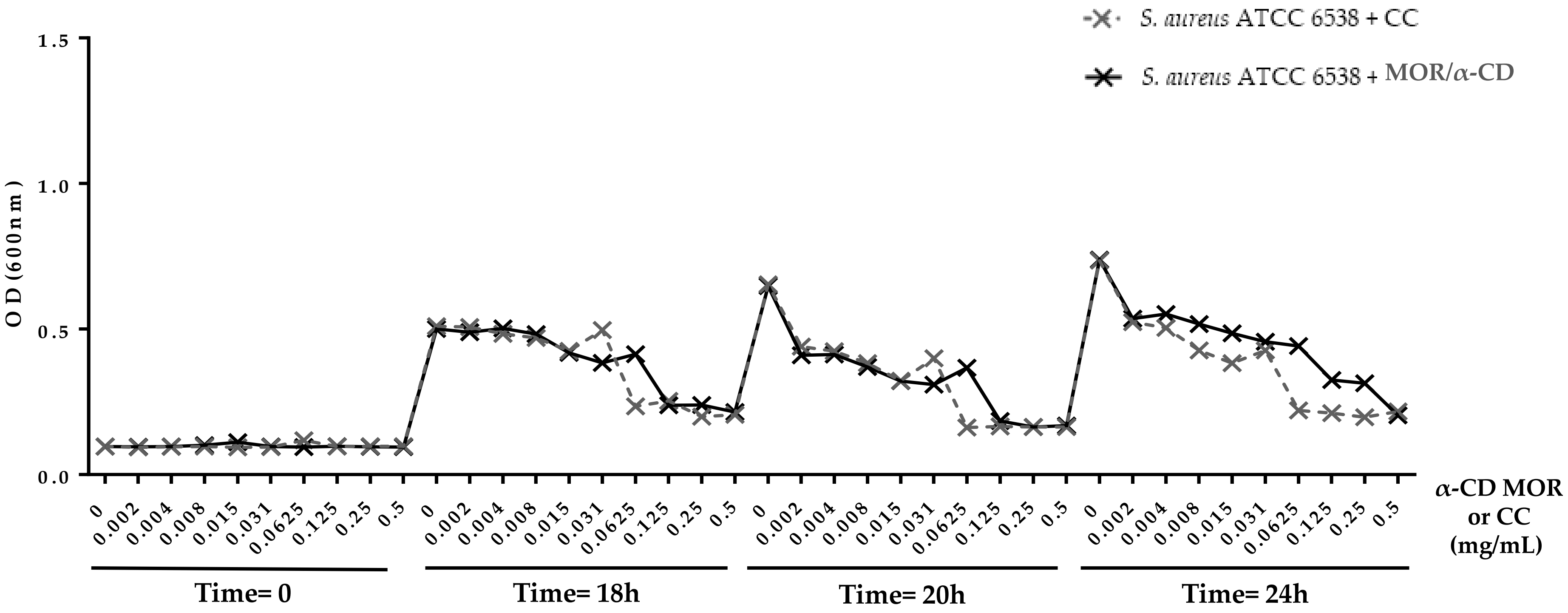

S3: MIC evaluation of MOR/α-CD against *S. aureus* ATCC 6538. The MIC value of MOR/α-CD was evaluated using a bacterial input of $5x10^5$ CFU/mL and measuring the optical density (OD) at time=0, time=18h, time=20h and time=24h. We assessed two fold dilution of MOR/α-CD (from 0,5 mg/mL to 0,002 mg/mL) compared to the same dilutions of clindamycin. The untreated bacteria (0 mg/mL of MOR/α-CD and 0 mg/mL of the clindamycin) represent the negative control.

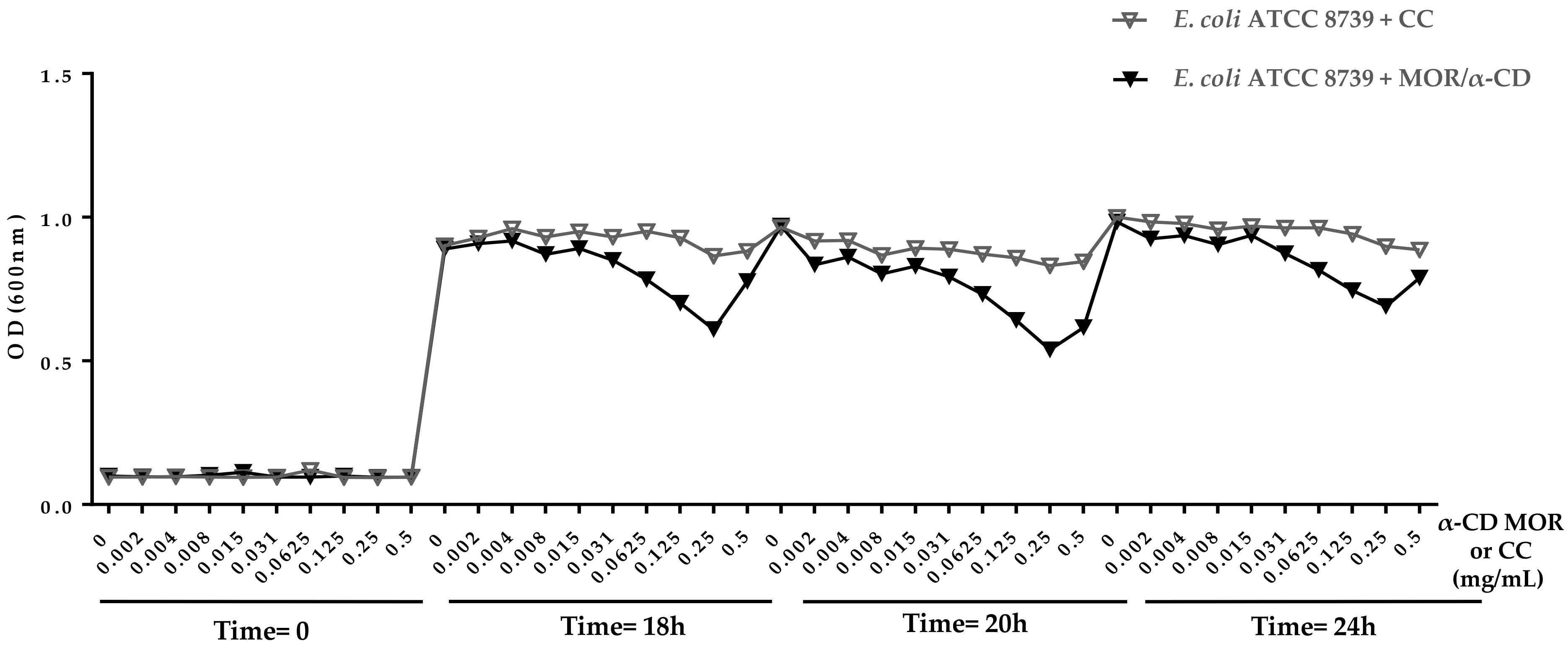

S4: MIC evaluation of MOR/α-CD against *E.coli* ATCC 8739. The MIC value of MOR/α-CD was evaluated using a bacterial input of $5x10^5$ CFU/mL and measuring the optical density (OD) at time=0, time=18h, time=20h and time=24h. We assessed two fold dilution of MOR/α-CD (from 0,5 mg/mL to 0,002 mg/mL) compared to the same dilutions of clindamycin. The untreated bacteria (0 mg/mL of MOR/α-CD and 0 mg/mL of the clindamycin) represent the negative control.

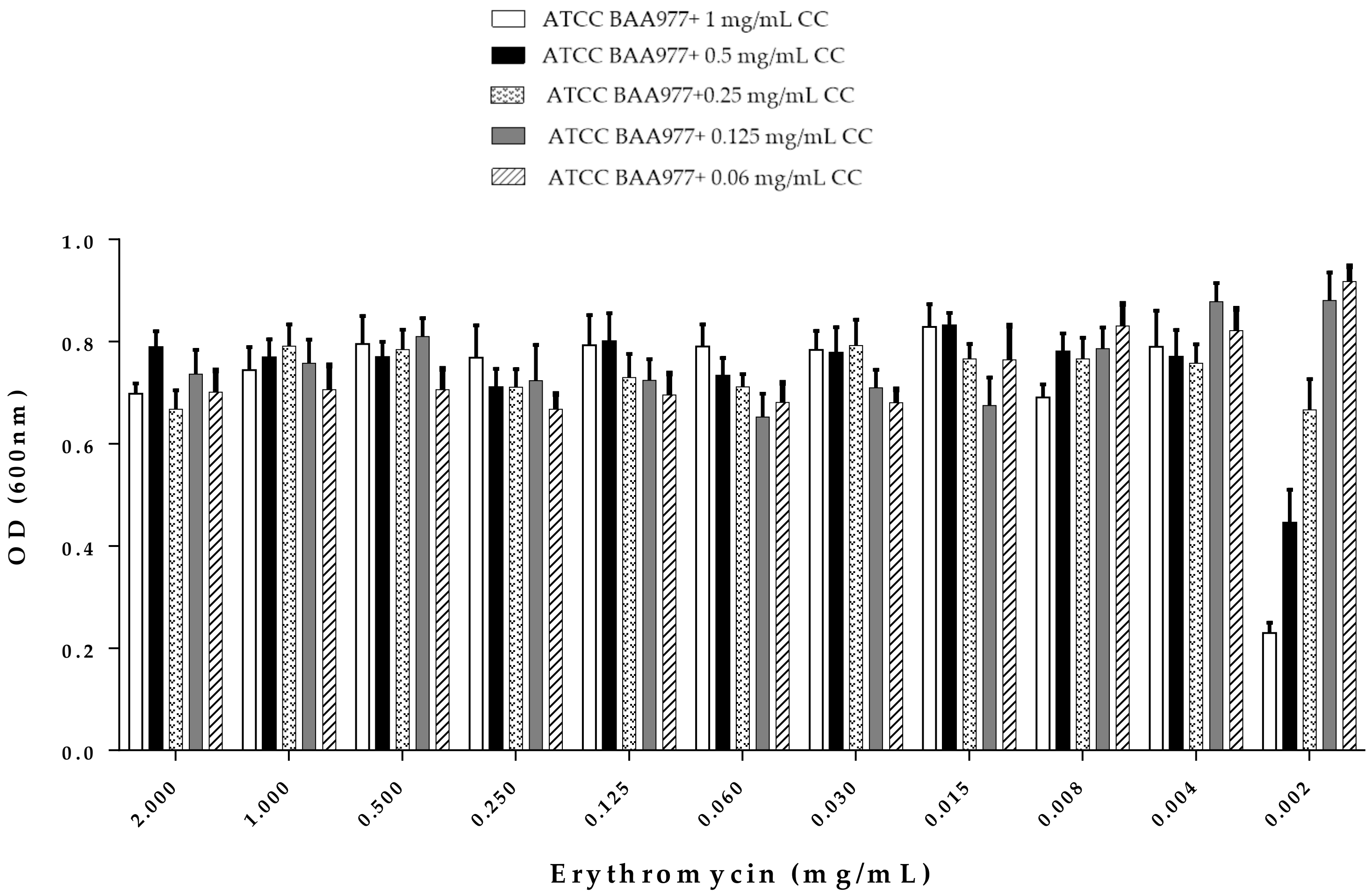

S5: Antibiotic combined assay to induce CC-resistance in the ATCC BAA-977 strain, carrying the *erm (A)* inducible CC-resistance gene. Experiment was performed using the doses of CC that naturally exerted antimicrobial activity against BAA-977 (1, 0.5, 0.25, 0.125, 0.06) combined with different doses of Ery (from 2 mg/mL to 0.002 mg/mL).

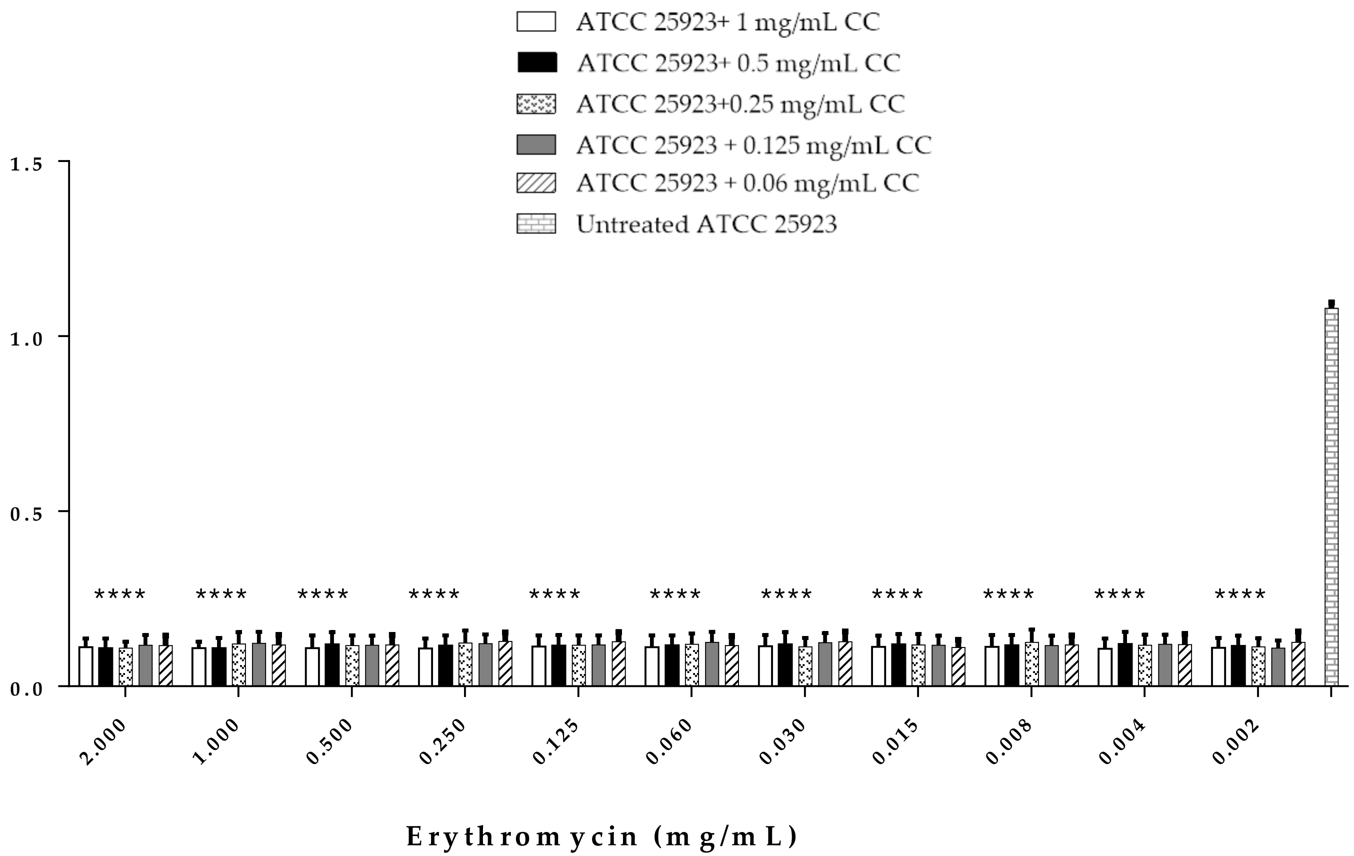

S6: Antibiotic combined assay against the ATCC 25923 lacking of resistance inducible gene. Experiment was performed using the doses of CC that exerted antimicrobial activity against ATCC 25923 (1, 0.5, 0.25, 0.125, 0.06 mg/mL) combined with different doses of Ery (from 2 mg/mL to 0.002 mg/mL). Experiments were assessed in triplicate to obtain the mean and the standard deviation (SD). Statistical analysis (Two way ANOVA) was performed by GraphPad Prism6 software and the P value < 0,0001 (****) showed significant difference between the treatment with 1 mg/mL of MOR/α-CD of the resistant strain in each combined well and the untreated bacteria (0 mg/mL).

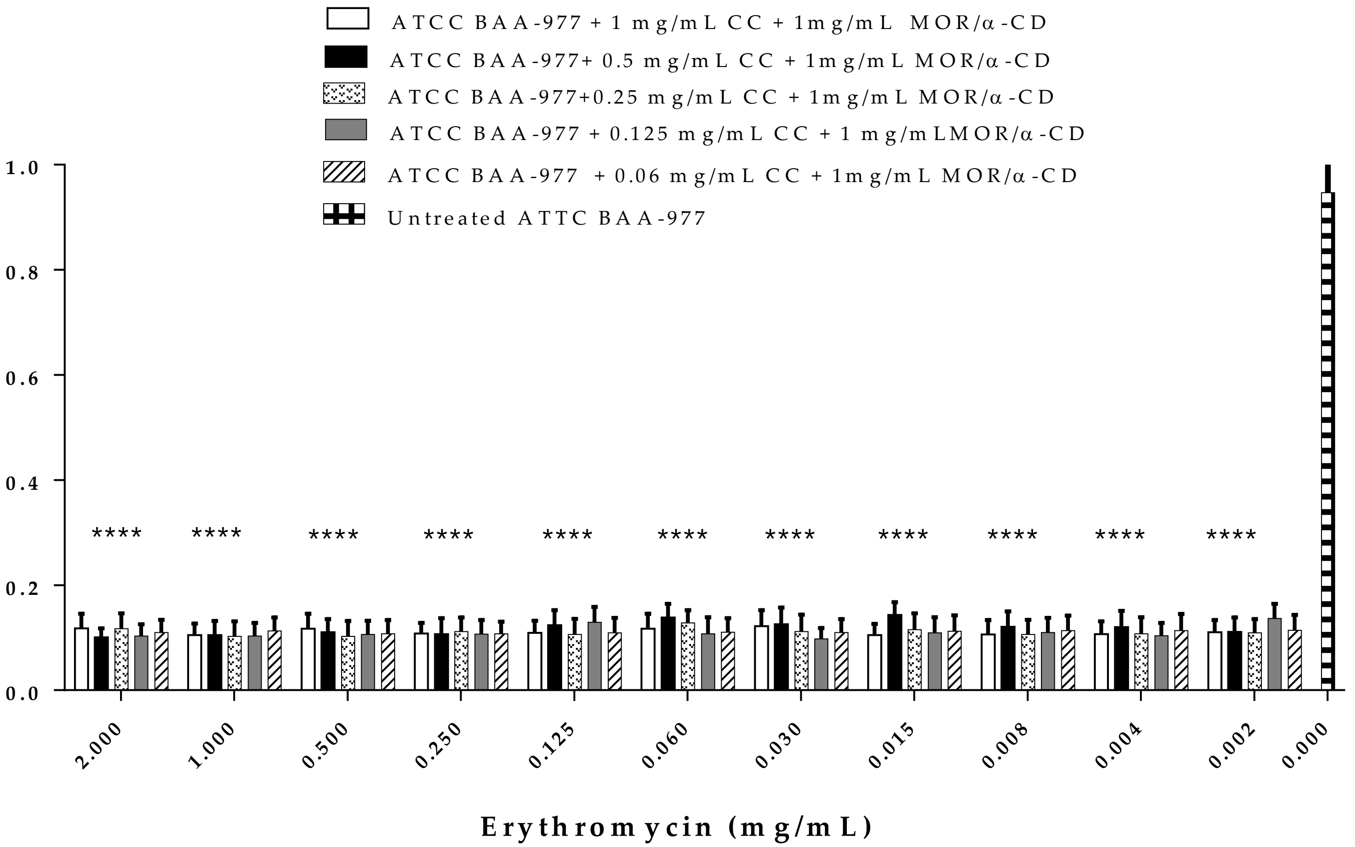

S7: MOR/α-CD growth inhibition activity against the ATCC BAA-977 CC-resistant strain. The experiment was carried out adding 1 mg/mL of MOR/α-CD for each microplate well, filled with the combined doses of CC (1, 0.5, 0.25, 0.125, 0.06 mg/mL) and Ery (from 2 mg/mL to 0.002 mg/mL). MOR/α-CD inhibition activity was evaluated by measuring the OD reached after 24h of treatment. Experiments were assessed in triplicate to obtain the mean and the standard deviation (SD). Statistical analysis (Two way ANOVA) was performed by GraphPad Prism6 software and the P value < 0,0001 (****) showed significant difference between the treatment with 1 mg/mL of MOR/α-CD of the resistant strain in each combined well and the untreated bacteria (0 mg/mL).
